# Blood platelets and serum VEGF in cancer patients

**DOI:** 10.1038/sj.bjc.6690059

**Published:** 1999-01


*British Journal of Cancer* (1999) **79**(2), 370–376
© 1999 Cancer Research Campaign

Letters to the Editor

### Blood platelets and serum VEGF in cancer patients

**Sir,**

We read with interest the publication of Banks et al (1998) on the significance of platelets for vascular endothelial growth factor (VEGF) measurements. We disagree, however, with the conclusion that for accurate measurement of circulating VEGF, serum is completely unsuitable. We are also convinced that platelets are not the sole origin of circulating VEGF. We would like to stress the potential clinical usefulness of the determination of serum VEGF levels in cancer patients based on published data and on some recent original observations. In the studies described below, the enzyme-linked immunosorbent assay (ELISA) methodology used by Banks et al (1998) was applied.

In 44 untreated patients with disseminated colorectal cancer, elevated serum VEGF, i.e. above 500 pg $ml^{-1}$, was significantly associated with fast tumour growth, i.e. estimated volume doubling time of less than 6 months (Dirix et al, 1996), independent of the number or site of metastasis, suggesting a predictive value of serum VEGF for the progression of the disease. In 42 treated metastatic cancer patients, including patients with colorectal, breast, ovarian and renal carcinomas, a significantly higher fraction, i.e. 32%, of patients with progressive disease during chemotherapeutic treatment had elevated serum VEGF compared with 15% of patients showing response to treatment (Dirix et al, 1997). In 82 patients with non-Hodgkin's lymphoma, followed up for at least 5 years, 71% of patients with lower than the median serum VEGF before treatment had a 5-year survival rate compared with only 49% among patients with a higher than the median serum VEGF ($P$ = 0.01) (Salven et al, 1997*a*). In small-cell-lung cancer ($n$ = 68), a high pretreatment serum VEGF predicts poor response to chemotherapy ($P$ = 0.008) and shorter survival ($P$ = 0.01) (Salven et al, 1998). The results of these and other clinical studies (Salven et al, 1997*b*; Vermeulen et al, 1997) suggest the potential relevance of measuring serum VEGF concentrations in cancer patients.

Because of the association of serum VEGF concentration and platelet count in 27 breast cancer patients reported by Verheul et al (1997) and the increase in VEGF concentration after activation of platelet-rich plasma reported by Banks et al (1998), we have compared the platelet count with serum VEGF and basic fibroblast growth factor (bFGF) levels in 58 patients (142 measurements). We found an association between platelet count and serum VEGF ($r$ = 0.50; 95% confidence interval 0.37–0.61; $P$ < 0.0001) (Figure 1), but not between platelet count and serum bFGF ($r$ = 0.06; $P$ = 0.5). In 12 patients for whom we had four or more blood samples at different times during treatment or follow-up, we analysed the intrapatient association of serum VEGF and platelet count. In 10 out of 12 patients (83%), the blood sample with the highest platelet count also had the highest serum VEGF level, although this was the case for only 5 out of 12 patients (42%) for serum bFGF. Changes in serum VEGF, i.e. increase or decrease, were congruent with changes in platelet count in 82% of 39 changes. For serum bFGF, this was the case for only 59% of all changes.

We argued that one of the reasons for the low coefficient of correlation ($r$-value) of the association between serum VEGF and platelet count might be the interindividual difference in the ratio between serum VEGF and platelet count. Of all 142 blood samples, the mean ratio of VEGF and platelet count was 1.39 pg VEGF per $10^6$ platelets (standard deviation 0.96; median 1.16; range 0.16–4.96). The coefficient of variation was, therefore, 69%. The individual coefficients of variation were much lower. Twenty-three patients, with three or more measurements, were analysed. Mean coefficient of variation was 26% (standard deviation 14; median 23; range 6–56), indicating that interindividual variation of the ratio between serum VEGF and the number of platelets is larger than intraindividual variation. A positive association between serum VEGF and the ratio between serum VEGF and platelet count was also noted ($r$ = 0.83; 95% confidence interval 0.78–0.88; $P$ < 0.0001). If, as can be concluded from the more than tenfold higher VEGF concentrations in serum compared with plasma and the invariably low levels of plasma VEGF in Table 2 of the publication of Banks et al (1998), the majority of circulating VEGF protein is associated with platelets, this means that the amount of VEGF per platelet is different from patient to patient. So, serum VEGF does not only reflect platelet count. Indeed, VEGF mRNA can also be detected in other cell fractions of the peripheral blood, including monocytes, granulocytes and lymphocytes (Wartiovaara et al, 1998).

The mitogenic activity on endothelial cells of the sera of 20 cancer patients was compared with that of fetal calf serum. The patients' sera with bFGF concentrations higher than the cut-off value which correlates with fast growth kinetics (Dirix et al, 1996), i.e. 7.5 pg $ml^{-1}$, stimulated human umbilical vein endothelial cell (HUVEC) proliferation significantly more, i.e. 1.7 times (1.3–2.3), than the control serum only when the VEGF concentration in the sera was also elevated, i.e. higher than 500 pg $ml^{-1}$. In sera with low-bFGF or low-VEGF content, no stimulation of HUVEC proliferation was observed. Therefore, we conclude that serum VEGF measurements in cancer patients correspond to the degree of mitogenic activity of the serum on endothelial cells.

Given the association of serum VEGF with the clinical course of cancer patients, the lack of a strong association between serum VEGF and platelet count, and the association of serum VEGF with the degree of stimulation of endothelial cell proliferation in vitro, we conclude that measuring serum VEGF might be more suitable in cancer patients than measuring plasma VEGF. Taking into account errors caused by sample handling and the fact that VEGF mRNA is also present in other blood cells, measuring VEGF in whole blood might even be more accurate. Recent results (P Salven, unpublished data) indeed suggest that using whole blood when measuring circulating VEGF may improve the clinical significance.

It is tempting to speculate on the mechanism responsible for the association between platelet count and serum VEGF. Although megakaryocytes produce VEGF (Möhle et al, 1997), in cancer patients another, and presumably important, source of circulating

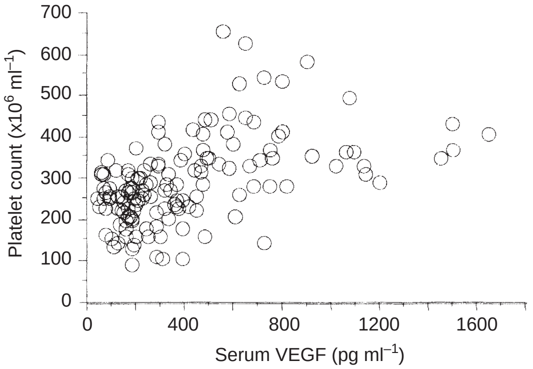

**Figure 1** Association of serum VEGF and platelet count in 58 cancer patients (142 samples) $r = 0.5$, $P < 0.0001$. Serum VEGF was analysed by ELISA (Quantikine human VEGF, R & D Systems, Minneapolis, MN, USA)

VEGF is the tumour. An interesting hypothesis is that in fast-growing tumours, in addition to up-regulation of VEGF, the production of a potent thrombopoietic cytokine, e.g. interleukin 6 (IL-6) (Ishibashi et al, 1989), would be increased. Indeed, like VEGF, IL-6 is up-regulated by hypoxia (Yan et al, 1995) and IL-6 expression is elevated in tissues that undergo active angiogenesis, such as healing wounds (Mateo et al, 1994). Moreover, response elements for IL-6 signalling have been found upstream to the transcription initiation site of VEGF, and treating cell lines with IL-6 resulted in induction of VEGF mRNA (Cohen et al, 1996). We have observed twofold higher serum VEGF levels in malignant mesothelioma patients compared with breast and colorectal cancer patients (Kumar-Singh et al, 1997). Among patients with malignant mesothelioma, a high incidence of thrombocytosis has been reported, which is associated with high levels of circulating IL-6 (Nakano et al, 1998). Benign angiogenic diseases are also characterized by thrombocytosis. For example, in inflammatory bowel disease, elevated serum levels of angiogenic factors and of IL-6, and high platelet count, are associated with exacerbation of disease (Lake et al, 1978; Bross et al, 1996; Bousvaros et al, 1997).

From a physiological point of view, platelets could, thus, be regarded as scavengers of circulating VEGF, in order to restrict the angiogenic activity to sites of wound healing, of growing organs, of inflammation and, in the case of cancer patients, of tumour progression. This mechanism would explain part of the contradiction between the high-angiogenic activity of cancer patients' serum and the lack of angiogenesis outside the tumour tissue. Activated platelets have indeed been found to release VEGF together with β-thromboglobulin, suggesting that VEGF resides in the α-granules of the platelets (Wartiovaara et al, 1998). This leaves open the possibility that at least part of the VEGF in platelets has been endocytosed from the plasma.

*PB Vermeulen[1], P Salven[3], I Benoy[1], G Gasparini[3] and LY Dirix[1]*
*[1]Angiogenesis Group, Experimental Oncology, Oncological Centre, AZ St-Camillus/St-Augustinus, Oosterveldlaan 24, B2610 Wilrijk-Antwerp, Belgium*; *[2]Department of Oncology, Helsinki University Central Hospital, Haartmaninkatu 4, 00290 Helsinki, Finland*; *[3]Division of Medical Oncology, Azienda Ospedaliera 'Bianchi-Melacrino-Morelli', Via Melacrino, 89100 Reggio Calabria, Italy*

### REFERENCES

Banks RE, Forbes MA, Kinsey SE, Stanley A, Ingham E, Walters C and Selby PJ (1998) Release of the angiogenic cytokine vascular endothelial growth factor (VEGF) from platelets: significance for VEGF measurements and cancer biology. *Br J Cancer* **77**: 956–964

Bousvaros A, Zurakowski D, Fishman SJ, Keough K, Law T, Sun C and Leichter AM (1997) Serum basic fibroblast growth factor in pediatric Crohn's disease. Implications for wound healing. *Dig Dis Sci* **42**: 378–386

Bross DA, Leichtner AM, Zurakowski D, Law T and Bousvaros A (1996) Elevation of serum interleukin-6 but not serum-soluble interleukin-2 receptor in children with Crohm's disease. *J Pediatr Gastroenterol Nutr* **23**: 164–171

Cohen T, Nahari D, Weiss Cerem L, Neufeld G and Levi B (1996) Interleukin 6 induces the expression of vascular endothelial growth factor. *J Biol Chem* **271**: 736–741

Dirix LY, Vermeulen PB, Hubens G, Benoy I, Martin M, De Pooter C and Van Oosterom AT (1996) Serum basic fibroblast growth factor and vascular endothelial growth factor and tumour growth kinetics in advanced colorectal cancer. *Ann Oncol* **7**: 843–848

Dirix LY, Vermeulen PB, Pawinski A, Prové A, Benoy I, De Pooter C, Martin M and Van Oosterom AT (1997) Elevated levels of the angiogenic cytokines basic fibroblast growth factor and vascular endothelial growth factor in sera of cancer patients. *Br J Cancer* **76**: 238–243

Ishibashi T, Kimura H, Shikama Y, Uchida T, Kariyone S, Hirano T, Kishimoto T, Takatsuki F and Akiyama Y (1989) Interleukin-6 is a potent thrombopoietic factor in vivo in mice. *Blood* **74**: 1241–1244

Kumar-Singh S, Vermeulen PB, Weyler J, Segers K, Weyn B, Van Daele A, Dirix LY, Van Oosterom AT and Van Marck E (1997) Evaluation of tumour angiogenesis as a prognostic marker in malignant mesothelioma. *J Pathol* **182**: 211–216

Lake AM, Stauffer JQ and Stuart MJ (1978) Hemostatic alterations in inflammatory bowel disease: response to therapy. *Am J Dig Dis* **23**: 897–902

Mateo RB, Reichner JS and Albina JE (1994) Interleukin-6 activity in wounds. *Am J Physiol* **266**: R1840–1844

Möhle R, Green D, Moore MAS, Nachman RL and Rafii S (1997) Constitutive production and thrombin-induced release of vascular endothelial growth factor by human megakaryocytes and platelets. *Proc Natl Acad Sci USA* **94**: 663–668

Nakano T, Chaninian AP, Shinjo M, Tonomura A, Miyake M, Togawa N, Ninomiya K and Higashino K (1998) Interleukin 6 and its relationship to clinical parameters in patients with malignant pleural mesothelioma. *Br J Cancer* **77**: 907–912

Salven P, Teerenhovi L and Joensuu H (1997*a*) A high pretreatment serum vascular endothelial growth factor concentration is associated with poor outcome in non-Hodgkin's lymphoma. *Blood* **90**: 3167–3172

Salven P, Mäenpää H, Orpana A, Alitalo K and Joensuu H (1997*b*) Serum vascular endothelial growth factor is often elevated in disseminated cancer. *Clin Cancer Res* **3**: 647–652

Salven P, Ruotsalainen, Mattson K and Joensuu H (1998) High pretreatment serum level of vascular endothelial growth factor (VEGF) is associated with poor outcome in small cell lung cancer. *Int J Cancer* **79**(2): 144–146

Yan SF, Tritto I, Pinsky D, Liao H, Huang J, Fuller G, Brett J, May L and Stern D (1995) Induction of interleukin 6 (IL-6) by hypoxia in vascular cells. *J Biol Chem* **270**: 11463–11471

Verheul HMW, Hoekman K, Luykx-de Bakker S, Eekman CA, Folman CC, Broxterman HJ and Pinedo HM (1997) Platelet: transporter of vascular endothelial growth factor. *Clin Cancer Res* **3**: 2187–2190

Vermeulen PB, Dirix LY, Martin M, Lemmens J and Van Oosterom AT (1997) The predictive value of serum bFGF and VEGF in patients with metastatic renal cell carcinoma treated with interferon α-2b. *J Natl Cancer Inst* **89**: 1317

Wartiovaara U, Salven P, Mikkola H, Lassila R, Kaukonen J, Joukov V, Orpana A, Ristimäki A, Heikinheimo M, Joensuu H, Alitalo K and Palotie A (1998) Peripheral blood platelets express VEGF-C and VEGF which are released during platelet activation. *Thromb Haemost* **80**(7): 171–173

© Cancer Research Campaign 1999 

### Release of the angiogenic cytokine vascular endothelial growth factor from platelets – reply to the letter from Vermeulen et al

**Sir,**

The letter by Vermeulen et al raises interesting possibilities about platelets and the measurement of circulating vascular endothelial growth factor (VEGF), several of which we alluded to in our original publication (Banks et al, 1998). It is now apparent that many haematological cells are capable of producing or releasing VEGF, including platelets, megakaryocytes, $CD34^+$ cells, monocytes, T-lymphocytes and neutrophils (Freeman et al, 1995; Katoh et al, 1995; Gaudry et al, 1997; Möhle et al, 1997; Verheul et al, 1997; Banks et al, 1998). However, the extent to which these cells normally contribute to the circulating VEGF pool in vivo and the physiological or pathological relevance is not yet clear.

The measurement of VEGF in citrated plasma under the descriptions described in our study (Banks et al, 1998) is most likely to reflect the true circulating VEGF concentration, i.e. any soluble VEGF which is not cell associated. The basic concept behind the measurement of systemic concentrations of cytokines is that it reflects localized events in particular organs because of an ‘overspill’ effect. In the case of VEGF, the hope is that the increased production of VEGF within a tumour is reflected by an increased circulating concentration, which may allow its use as a marker of angiogenesis. Clearly, if there is a constitutive production of any cytokine, this will form the basis for the baseline levels of cytokine measured in the absence of any disease, and will depend on the rate of production and entry into the circulation and its subsequent removal. Therefore, production of VEGF by tissues undergoing physiological angiogenesis, for example endometrium, is most likely to determine the normal circulating levels. Platelets are capable of producing VEGF endogenously with the demonstration of mRNA within platelets and megakaryocytes and the secretion of VEGF by both platelets and megakaryocytes after activation, but occurring constitutively only in megakaryocytes (Katoh et al, 1995; Mohle et al, 1997). Therefore, under normal circumstances, platelets may be expected to contribute minimally to the circulating concentrations of VEGF. The main exception to this would be, however, if platelets are serving as a ‘scavenger’ of circulating VEGF, as proposed by Vermeulen et al, and removing it from the circulation by endocytosis (for which there is as yet no published evidence). Then a dynamic equilibrium may exist in which their content varies depending on the production of VEGF as a result of the underlying disease process.

The measurement of serum concentrations of VEGF is an artificial situation and measures both the freely circulating VEGF and that released by platelets upon activation during the clotting process. Although other cell types, for example neutrophils, may also contribute to the serum VEGF, the significant correlation found in our study between platelet-derived VEGF in platelet-rich plasma in which other cell types are absent and theoretical platelet-derived VEGF in corresponding serum samples ($r = 0.95$, $P < 0.001$) makes it extremely likely that platelets are the main cellular source of serum VEGF (Banks et al, 1998). The fact that more recent studies are now demonstrating significant relationships between serum VEGF concentrations and clinical indices, as discussed by Vermeulen et al, is clearly of considerable biological interest. This may arise because of the proposed ‘scavenger’ function of platelets in endocytosing VEGF, but also equally may partly reflect platelet number which changes in many disease states including cancer. In support of this latter possibility, cyclical changes in serum VEGF concentration were found during chemotherapy, coinciding with the chemotherapy-induced thrombocytopenia and subsequent rebound increase in platelet number, rather than a sustained decrease in VEGF levels which would be expected if the tumour-derived VEGF was to decrease as a result of chemotherapy (Verheul et al, 1997). In agreement with the much larger study described by Vermeulen et al, we found marked interindividual variation in platelet VEGF content (Figure 2, Banks et al, 1998) and agree that differences in platelet content of VEGF as well as platelet number can also markedly influence serum VEGF concentration. Another point to bear in mind, particularly when examining correlations between platelet number and serum VEGF, is that serum VEGF concentrations are determined by the platelet-derived VEGF and circulating VEGF levels, and that if the latter increases with disease state (as can be determined from measurements using plasma) then this can become an increasingly important component of the serum VEGF concentration. This may account for the relatively low correlation coefficients between platelet number and serum VEGF. It may be more accurate to examine the correlation between platelet number and VEGF concentration measured as the difference between serum and plasma levels.

It is evident that further research is needed to investigate further the role of platelets in both physiological and pathological situations involving VEGF, and into whether serum measurements are providing clinically useful information only owing to the reflection of platelet numbers or for the other reasons discussed. We are currently carrying out a preliminary study examining the relationship between circulating plasma and serum VEGF concentrations, platelet number and the tissue expression of VEGF in patients with breast cancer which may provide some answers, but clearly other major studies are needed.

*RE Banks[1], MA Forbes[1], SE Kinsey[2], A Stanley[1], E Ingham[3], C Walters[3] and PJ Selby[1]*
*[1]ICRF Cancer Medicine Research Unit, St James's University Hospital, Leeds LS9 7TF, UK; [2]Department of Haematology, St James's University Hospital, Leeds LS9 7TF, UK; and [3]Department of Microbiology, University of Leeds, Leeds LS2 9JT, UK*

### REFERENCES

Banks RE, Forbes MA, Kinsey SE, Stanley A, Ingham E, Walters C and Selby PJ (1998) Release of the angiogenic cytokine vascular endothelial growth factor (VEGF) from platelets: significance for VEGF measurements and cancer biology. *Br J Cancer* **77**: 956–964

 *© Cancer Research Campaign 1999*

Gaudry M, Brgerie O, Andrieu V, El Benna J, Pocidalo MA and Hakim J (1997) Intracellular pool of vascular endothelial growth factor in human neutrophils. *Blood* **90**: 4153–4161
Möhle R, Green D, Moore MAS, Nachman RL and Rafii S (1997) Constitutive production and thrombin-induced release of vascular endothelial growth factor by human megakaryocytes and platelets. *Proc Natl Acad Sci USA* **94**: 663–668
Verheul HMW, Hoekman K, Luykx-de Bakker S, Eekman CA, Folman CC, Broxterman HJ and Pinedo HM (1997) Platelet: transporter of vascular endothelial growth factor. *Clin Cancer Res* **3**: 2187–2190
Freeman MR, Schneck FX, Gagnon ML, Corles C, Soker S, Niknejad K, Peoples GE and Klagsbrun M (1995) Peripheral blood T lymphocytes and lymphocytes infiltrating human cancers express vascular endothelial growth factor: a potential role for T cells in angiogenesis. *Cancer Res* **55**: 4140–4145
Katoh O, Tauchi H, Kawaishi K, Kimura A and Satow Y (1995) Expression of the vascular endothelial growth factor (VEGF) receptor gene, KDR, in hematopoietic cells and inhibitory effect of VEGF on apoptotic cell death caused by ionizing radiation. *Cancer Res* **55**: 5687–5692

### High incidence of Kaposi's sarcoma in Iceland and the Faroe Islands

**Sir,**

In a recent paper, Hjalgrim et al (1998) reported a surprisingly high incidence of Kaposi's sarcoma (KS) in Iceland and the Faroe Islands. No explanation for the high incidence rates of KS in these two North Atlantic communities was identified.

Soil parent materials of Iceland and the Faroe Islands are mainly extrusive igneous rocks, which are characterized by the presence of mafic (iron oxide-rich) minerals (Krauskopf, 1979). These minerals are known to be highly weatherable (Ollier, 1984), allowing a significant release of iron compounds into the environment, such as in vegetables. Interestingly, a similar magmatic substrate is present in the Mediterranean area (e.g. Sicily, Sardinia) and in subtropical Africa; both areas are known to exhibit high incidence rates of KS. In this connection, a previous study (Ziegler, 1993) found that African endemic KS shows a distribution that conforms geographically to the iron oxide-rich volcanic clays of the East African Rift System. We, therefore, suggest that some environmental factors related to the presence of melanocratic rocks (mafic minerals) might be involved in the high incidence rates of KS in Iceland and the Faroe Islands.

*T Simonart[1], JP Van Vooren[2], J Herbauts[3] and JR Boelaert[4]*
*[1]Departments of Dermatology and [2]Internal Medicine, Erasme University Hospital, B-1070 Brussels, Belgium; [3]Laboratory of Plant Genetics and Ecology, Free University of Brussels, B-1160 Brussels; [4]Department of Infectious Diseases, Algemeen Ziekenhuis Sint Jan, 8000 Brugge, Belgium*

### REFERENCES

Hjalgrim H, Tulinius H, Hardarson S, Frisch M and Melbye M (1998) High incidence of classical Kaposi's sarcoma in Iceland and the Faroe Islands. *Br J Cancer* **77**: 1190–1193
Krauskopf KB (1979) *Introduction to Geochemistry*. McGraw-Hill, Kogakusha: London
Ollier C (1984) *Weathering*. Longman Group: Essex
Ziegler JL (1993) Endemic Kaposi's sarcoma in Africa and local volcanic soils. *Lancet* **342**: 1348–1351

### Survival and determinants of response to third-line chemotherapy in sensitive recurrent ovarian cancer patients

**Sir,**

More than 70% of ovarian cancer patients achieve a partial or complete response to first-line chemotherapy (McGuire et al, 1996; Bolis et al, 1997). Most of them, however, will recur (Neijet et al, 1991; Bolis et al, 1996). The results of second-line treatment depend largely on the platinum-free interval (Markman et al, 1991), patients who relapse 6 months after primary chemotherapy having a higher response rate to platinum treatments (Thigpen et al, 1993; Bolis et al, 1994*a*). Unfortunately, the response is relatively short lasting, and most patients need a third-line chemotherapy.

Data on response to third-line chemotherapy in ovarian cancer patients responding to prior treatments are lacking. We analysed the response to third-line chemotherapy in a series of women responsive to first- and second-line therapy for ovarian cancer. They were observed between 1984 and 1997 in a network of centres collaborating in three randomized trials on second-line therapy for recurrent advanced ovarian cancer ≥ 6 months after first-line chemotherapy (Zanaboni et al, 1991; Bolis et al, 1994*a*, 1994*b*). As of June 1997, 56 out of 223 cases included in these trials had a second relapse after complete or partial response to second-line chemotherapy, including platinum-based compounds alone or in combination. Of these, 49 (median age 51 years, range 30–70) received third-line chemotherapy; these women are considered in this report.

Histological types at first diagnosis were: serous in 36 cases (74%), endometrioid in seven (14%) and others in six (12%). Grading was: (1) in two patients (4%); (2) in 14 (29%); and (3) in 33 (67%). Response to second-line treatment was complete in 31 patients (63%) and partial in 18 (37%) with a median duration of 10 months (range 3–60). A total of 13 (27%) relapsed within 6 months from second-line treatment and 36 (73%) after 6 months or more.

© Cancer Research Campaign 1999

Third-line chemotherapy regimens included topotecan or platinum compounds alone or platinum compounds in combination with anthracyclines or taxanes.

No women were lost to follow-up (median follow-up 11 months, range 2–53). Survival and response data were updated to August 1997.

Forty-seven women were evaluated for response to third-line chemotherapy, two patients did not complete planned treatments. Complete and partial response was observed, respectively, in 12 (27%) and in ten (21%) cases (Table 1). Overall response rate was 49%, with a median duration of 6 months (range 1–23).

A significant difference in 1-year survival was observed between responders (82%) and non-responders (39%, $P < 0.05$). Median survival was 16 months (range 4–53) in the first group and 9 months (range 1–31) in the second.

There were no differences in response according to patients' characteristics at first diagnosis. The response rate tended to be higher in patients with a complete remission (52%) to second-line treatment than those who had a partial one (37%), though the difference was not significant (Table 1).

Time to progression from last treatment, site of second relapse and type of third-line chemotherapy regimen seemed to have no marked impact on response, but we observed a higher response rate in patients who relapsed 6 months or more after salvage therapy (50%) than those who recurred before 6 months (38%); again, the difference was not significant.

Up to now, ovarian cancer patients were treated with third-line chemotherapy in phase I and II studies to test new agents and combinations (Eisenhauer et al, 1994; Kaye et al, 1995), but few studies have evaluated the determinants of response, which may be useful to identify patients who can benefit from third-line treatment. Our study found that about half of relapsing patients responsive to first- and second-line chemotherapy could benefit from third-line chemotherapy, though the response was short lasting. The frequency of response seems to be higher in patients who obtained complete remission to prior treatments. In the next few years, these findings may be useful with the introduction of novel antineoplastic drugs that will permit new therapeutic options.

**Table 1** Response and 1 year survival to third-line chemotherapy according to selected characteristics relapse

| | Response | |
|---|---|---|
| | Yes (%)[a] | No (%) |
| Total | 22 (49) | 23 (51) |
| Characteristic at diagnosis | | |
| Age | | |
| ≤55 | 14 (44) | 18 (56) |
| >55 | 8 (53) | 7 (47) |
| Grade | | |
| 1 | 2 (100) | – (0) |
| 2 | 3 (25) | 9 (75) |
| 3 | 17 (52) | 16 (48) |
| Histotype | | |
| Serous | 15 (43) | 20 (57) |
| Endometrioid | 4 (67) | 2 (33) |
| Others | 3 (50) | 3 (50) |
| Response to second-line chemotherapy | | |
| Complete | 16 (52) | 15 (48) |
| Partial | 6 (37) | 10 (63) |
| Time to progression after second-line chemotherapy (months) | | |
| <6 | 5 (38) | 8 (62) |
| ≥6 | 17 (50) | 17 (50) |
| Site of second relapse | | |
| Pelvic/abdominal | 18 (49) | 19 (51) |
| Extra-abdominal | 4 (40) | 6 (60) |
| Third-line chemotherapy regimens | | |
| Monochemotherapy | 12 (44) | 15 (56) |
| Polychemotherapy | 10 (50) | 10 (50) |

[a]Complete or partial.

*A Villa[1], F Parazzini[2], G Scarfone[1,2,3], P Guarnerio[2] and G Bolis[1]*
*[1]Istituto Tumori, Divisione Ginecologia Oncologica, Milano, Italy; [2]Prima Clinica Ostetrico Ginecologica, Università di Milano, Milano, Italy; [3]Istituto Mario Negri, Milano, Italy*

### REFERENCES

Bolis G, Scarfone G, Luchini L, Ferraris C, Zanaboni F, Presti M, Giardina G, Villa A and Parazzini F (1994*a*) Response to second-line weekly cipslatin chemotherapy in ovarian cancer previously treated with a cisplatin- or carboplatin-based regimen. *Eur J Cancer* **30**: 1764–1768

Bolis G, Brusati M, Ferraris C, Franchi M, Maggi R, Merisio C, Scarfone G, Villa A, Zanaboni F and Ferrari A (1994*b*) Carboplatin alone (c) vs carboplatin + high-dose epirubicin (HDCE) + growth-factors (GF) in late recurrences ovarian cancer (PTS). *Proc Am Soc Clin Oncol* **869**: 271

Bolis G, Villa A, Guarnerio P, Ferraris C, Gavoni N, Giardina G, Melpignano M, Scarfone G, Zanaboni F and Parazzini F (1996) Survival of women with advanced ovarian cancer and complete response at second-look laparotomy. *Cancer* **77**: 128–131

Bolis G, Scarfone G, Zanaboni F, Villa A, Guarnerio P, Gentile A, Presti M, Melpignano M, Ferraris C, Tateo S and Parazzini F (1997) A phase I–II trial of fixed-dose carboplatin and esclating paclitaxel in advanced ovarian cancer. *Eur J Cancer* **33**: 592–595

Eisenhauer EA, ten Bokkel Huinink WW, Swenerton KD, Gianni L, Myles J, van der Burg MEL, Kerr I, Vermorken JB, Buser K, Colombo N, Bacon M, Santabárbara P, Onetto N, Winograd B and Canetta R (1994) European–Canadian randomized trial of paclitaxel in relapsed ovarian cancer. High-dose versus low-dose and long versus short infusion. *J Clin Oncol* **12**: 2654–2666

Kaye SB, Piccart M, Aapro M and Kavenagh J (1995) Docetaxel in advanced ovarian cancer: Preliminary results from three phase II trials. *Eur J Cancer* **31**: 514–517

Markman M, Rothman R, Hakast T, Reichman B, Hoskins W, Rubin S, Jones W, Almadrones L and Lewis JL (1991) Second-line platinum therapy in patients with ovarian cancer previously treated with cisplatin. *J Clin Oncol* **9**: 389–393

McGuire WP, Hoskins WJ, Brady MF, Kucera PR, Partridge EE, Look KY, Clarke-Pearson DL and Davidson M (1996) Cyclophosphamide and cisplatin compared with paclitaxel and cisplatin in patients with stage III and stage IV ovarian cancer. *N Engl J Med* **334**: 1–6

Neijet JP, ten Bokkel Huinink WW, van der Burg MEL, van Oosterom A, Willemse P and Vermorken J (1991) Long-term survival in ovarian cancer: mature data from The Netherlands Joint Study Group for ovarian cancer. *Eur J Cancer* **27**: 1367–1372

Thigpen JT, Vance RB and Khansur T (1993) Second-line chemotherapy for recurrent carcinoma of the ovary. *Cancer* **71**: 1559–1564

Zanaboni F, Scarfone G, Presti M, Maggi R, Borello C and Bolis G (1991) Salvage chemotherapy for ovarian cancer recurrence: weekly cisplatin in combination with epirubicin or etoposide. *Gynecol Oncol* **43**: 24–28

 *© Cancer Research Campaign 1999*

### Inhibition of proteasome, apoptosis and sensitization to tumour necrosis factor alpha: do they always go together?

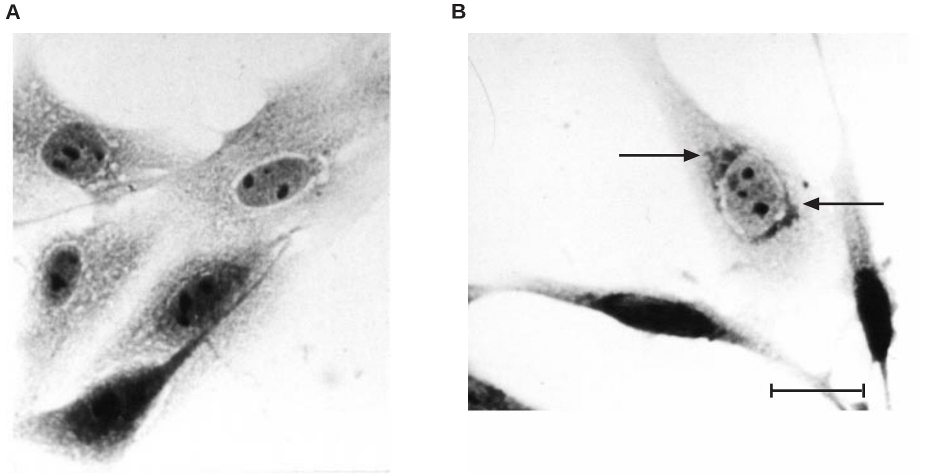

**Figure 1** B16F10 melanoma cells untreated (**A**) and treated with 5 μM PSI for 24 h (**B**), stained with amido black 10B. Arrows indicate perinuclear protein conjugates accumulated upon proteasome inhibition. Two other treated cells already display chromatin condensation. Bar = 20 μm

**Sir,**

A recent report by Delic et al (1998) showed that lactacystin sensitizes human leukaemia cells to the proapoptotic action of tumour necrosis factor alpha (TNF-α). This effect was attributed to the inhibition of the proteasome, assuming that lactacystin is a specific inhibitor of the latter. However, in a recent report (Ostrowska et al, 1997), it was shown that lactacystin is also capable of inhibiting the carboxypeptidase activity of cathepsin A. Therefore, any conclusions regarding the mechanism of action of lactacystin, as due to the inhibition of the proteasome alone, should be accepted only with caution. In another report (Wójcik et al, 1997), we have shown that a selective proteasome inhibitor of a different type (peptidyl-aldehyde) – PSI – induces apoptosis of L1210 murine leukaemia cells. Incubation of PSI-treated cells with 10 ng $ml^{-1}$ or 100 ng $ml^{-1}$ human TNF-α did not result in any increased rates of cell death, whereas TNF-α alone induced a measurable effect. As PSI also inhibits calpains, Z-leu-leucinal (ZLL), a calpain inhibitor without effects on the proteasome, was used as a control.

A similar approach was used to another cell line, B16F10 murine melanoma. Our experiments revealed that B16F10 melanoma cells cultured in vitro are very sensitive to PSI, which induces apoptotic changes and characteristic concentration of protein conjugates (Figure 1) in nanomolar concentrations although ZLL does not. PSI was dissolved in DMSO and no apoptotic effect was observed in cell cultures incubated with DMSO alone (the final solution in culture medium was less than 0.5%). The cytotoxic/cytostatic effect of PSI was dose dependent (Figure 2). TNF-α alone moderately inhibited the proliferation of melanoma cells. Only at the lowest PSI dose (5 nM) has there been an additive apoptosis promoting effect of TNF-α with PSI. At higher PSI doses, there was no difference if TNF-α was added to the culture or not, regardless of the time at which it was added to cell cultures (simultaneously or 1 or 2 days after PSI; data not shown).

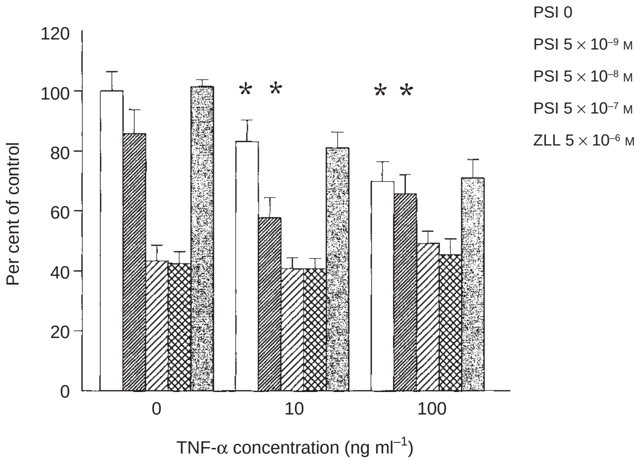

**Figure 2** Comparison of cytostatic/cytotoxic effects toward B16F10 melanoma cells in the absence or presence of increasing PSI or ZLL and TNF-α concentrations after 72 h incubation. Relative viability, expressed as per cent of control, was measured by the MTT assay, as described previously (Lasek et al, 1996). Standard deviations are indicated by vertical bars. The groups which differed significantly from control ($P < 0.05$, Student's *t*-test) are indicated by asterisks

It seems that suppression of NF-κB activation may be crucial to allow proapoptotic compounds to exert their effect (Wang et al, 1996), but as we have shown these result may be limited only to certain conditions and certain cell types.

© Cancer Research Campaign 1999

*T Stokłosa[1], C Wójcik[2], J Gołąb[1], A Giermasz[1] and S Wilk[3]*
*[1]Department of Immunology, Institute of Biostructure, Warsaw Medical University, Chałubińskiego 5, 02-004 Warsaw, Poland; [2]Department of Histology and Embryology, Institute of Biostructure, Warsaw Medical University, Chałubińskiego 5, 02-004 Warsaw, Poland; [3]Department of Pharmacology, Mount Sinai School of Medicine, City University of New York, New York, NY 10029, USA*

### ACKNOWLEDGEMENTS

J Gołąb is a recipient of the Foundation for Polish Science Award. This research was supported by grant no. 6 P207 058 07 from the State Committee for Scientific Research.

### REFERENCES

Delic J, Masdehors P, Omura S, Cosset J-M, Dumont J, Binet J-L and Magdelenat H (1998) The proteasome inhibitor lactacystin induces apoptosis and sensitizes chemo- and radioresistant human chronic lymphocytic leukaemia lymphocytes to TNF-alpha-initiated apoptosis. *Br J Cancer* **77**: 1103–1107

Lasek W, Giermasz A, Kuc K, Wańkowicz A, Feleszko W, Gołąb J, Zagożdżon R, Stoklosa T and Jakóbisiak M (1996) Potentiation of the anti-tumor effect of actinomycin D by tumor necrosis factor alpha in mice: correlation between in vitro and in vivo results. *Int J Cancer* **66**: 374–379

Ostrowska H, Wójcik C, Omura S and Worowski K (1997) Lactacystin, a specific inhibitor of the proteasome, inhibits human platelet lysosomal cathepsin A-like enzyme. *Biochem Biophys Res Commun* **234**: 729–732

Wang C-Y, Mayo MW and Baldwin Jr AS (1996) TNF- and cancer therapy-induced apoptosis: potentiation by inhibition of NF-κB. *Science* **274**: 784–787

Wójcik C, Stokłosa T, Giermasz A, Gołąb J, Zagożdżon R, Kawiak J, Wilk S, Komar A, Kaca A, Malejczyk J and Jakóbisiak M (1997) Apoptosis induced in L1210 leukaemia cells by an inhibitor of the chymotrypsin-like activity of the proteasome. *Apoptosis* **2**: 455–462

*© Cancer Research Campaign 1999*

